# Clinical Impact of Tumor-Infiltrating Inflammatory Cells in Primary Small Cell Esophageal Carcinoma

**DOI:** 10.3390/ijms15069718

**Published:** 2014-05-30

**Authors:** Yuling Zhang, Hongzheng Ren, Lu Wang, Zhifeng Ning, Yixuan Zhuang, Jinfeng Gan, Shaobin Chen, David Zhou, Hua Zhu, Dongfeng Tan, Hao Zhang

**Affiliations:** 1 Department of Information, Affiliated Cancer Hospital of Shantou University Medical College, Shantou 515031, China; E-Mail: zyl88900406@126.com; 2 Department of Pathology, the Central Hospital of Kaifeng, Kaifeng 475000, China; E-Mail: 13683789899@163.com; 3 Department of Biotherapy and Gastrointestinal Medical Oncology, Affiliated Cancer Hospital of Shantou University Medical College, Shantou 515031, China; E-Mails: lullawang@163.com (L.W.); ningzhifeng1976@163.com (Z.N.); jinfenggan@163.com (J.G.); 4 Department of Thoracic Surgery, Affiliated Cancer Hospital of Shantou University Medical College, Shantou 515031, China; E-Mail: chensb535176@hotmail.com; 5 Department of Pathology, University of Rochester Medical Center, Rochester, NY 14642, USA; E-Mail: david_zhou@urmc.rochester.edu; 6 Department of Surgery, Davis Heart and Lung Research Institute, Ohio State University Wexner Medical Center, Columbus, OH 43210, USA; E-Mail: hua.zhu@osumc.edu; 7 Department of Pathology, the University of Texas MD Anderson Cancer Center, Houston, TX 77030, USA; E-Mail: dongfengtan@yahoo.com; 8 Cancer Research Center, Shantou University Medical College, Shantou 515031, China; 9 Tumor Tissue Bank, Affiliated Cancer Hospital of Shantou University Medical College, Shantou 515031, China; E-Mail: zhyixuan@126.com

**Keywords:** primary small cell esophageal carcinoma, tumor associated macrophages, tumor associated eosinophils, prognosis, tumor infiltrating lymphocytes, esophageal squamous cell carcinoma

## Abstract

Primary small cell esophageal carcinoma is a rare and aggressive type of gastrointestinal cancer with poor prognosis. In the present study, the impact of tumour infiltrating inflammatory cells on clinico-pathological characteristics and the patients’ prognosis were analysed. A total of 36 small cell esophageal carcinomas, 19 adjacent normal tissues and 16 esophageal squamous cell carcinoma samples were collected. Qualified pathologists examined eosinophils, neutrophils, lymphocytes and macrophages on histochemical slides. The infiltration of eosinophils and macrophages in small cell esophageal carcinoma was significantly increased as compared with tumor adjacent normal tissues, and was significantly less in esophageal squamous cell carcinoma. Macrophage count was significantly associated with (*p* = 0.015) lymph node—stage in small cell esophageal carcinoma. When we grouped patients into two groups by counts of infiltrated inflammatory cells, Kaplan-Meier analysis revealed that high macrophage infiltration group (*p* = 0.004) and high eosinophil infiltration group (*p* = 0.027) had significantly enhanced survival. In addition, multivariate analysis unveiled that eosinophil count (*p* = 0.002) and chemotherapy (Yes *vs.* No, *p* = 0.001) were independent prognostic indicators. Taken together, infiltration of macrophages and eosinophils into the solid tumor appear to be important in the progression of small cell esophageal carcinoma and patients’ prognosis.

## 1. Introduction

Primary small cell esophageal carcinoma (SmCEC) is a rare but aggressive type of neuroendocrine malignancy affecting the esophagus in the gastrointestinal tract. SmCEC is characterized by rapid progression, spreads to distant sites early and has a dismal prognosis. No standard treatment has been established and few studies report long-term survival of patients [1,2,3]. Since, SmCEC is histologically similar to small-cell lung cancer; therapies for small-cell lung cancer are usually used to treat SmCEC. Most commonly used agents are cisplatin, etoposide, cyclophosphamide and doxorubicin and these agents are usually used in combination [4]. 

A growing body of evidence supports the notion that crosstalk between cancer cells and inflammatory cells in the tumor microenvironment influence the tumor development, progression, and resistance to radio-chemotherapy and hence the clinical outcome. Carcinogenesis of the esophagus is usually associated with inflammation [5,6,7,8,9], in which inflammatory signaling is activated and as a result, inflammatory cells are aberrantly infiltrated [10,11,12,13,14,15,16]. Lindau *et al.* [17,18,19] reported that the immune system of the tumor bearing host interacts with the tumor throughout its development and hence, this feature serve as a therapeutic target for anti-cancer immunotherapy. A high degree of inflammation was observed in human esophageal cancers [5,8,20,21], animal models of esophageal cancer [22], and esophageal cancer cell lines [23], and this inflammation plays a role in the carcinogenesis of esophageal tumors [5,20,21,24]. Although inflammatory cells have been studied in esophageal squamous cell carcinoma (ESqCC) [10,13,16], esophageal adenocarcinoma [11,25], and other small cell cancers [26], no such information is known in SmCEC. A meta analysis acknowledged that the prognostic value of inflammatory cells vary depending on the histological type of the cancer [27]; prognosis differs between the two histological subtypes, ESqCC and esophageal adenocarcinoma [28,29,30]. It is well known that SmCEC is similar to small cell lung cancer [31], and different from ESqCC and esophageal adenocarcinoma in tumor histology. Despite the presumed importance, the role of tumor infiltrating inflammatory cells in SmCEC has not been previously examined. One of the reasons for lack of studies on SmCEC is that SmCEC is a rare disease compared to the other two forms of esophageal cancer and sample sizes have been usually less in the published literature with most of them in the form of case reports. 

Thus, our aim in this study was to investigate the prognostic influence and relationship between tumor infiltrating inflammatory cells and various clinico-pathological characteristics of SmCEC patients with a slightly large sample size from two high incidence areas in China.

## 2. Results and Discussion

### 2.1. Patients’ Demographics, Clinico-Pathological Characteristics and Treatment

In this study, 25 men and 11 women with an age range from 45 to 77 (median age 59 years) constituted the total sample size. The majority of tumors in 23 out of 36 patients (63.9%) were found to be present in the middle third of the esophagus. All the 36 patients underwent transthoracic esophagectomy with two-field lymphadenoectomy. Of the 36 patients, 24 patients received surgical treatment alone; nine were treated with surgery followed by postoperative chemotherapy and three patients were treated with surgery followed by a combination of postoperative radio-chemotherapy. Tumor recurrence and metastasis to the adjacent organs (frequently to the liver) were observed in nine patients. Clinico-pathologic characteristics of the patients are presented in Table S1.

### 2.2. Eosinophils and Macrophages Are Increased in SmCEC (Small Cell Esophageal Carcinoma) Tissues in Comparison with Tumor Adjacent Normal Tissues

**Figure 1:**
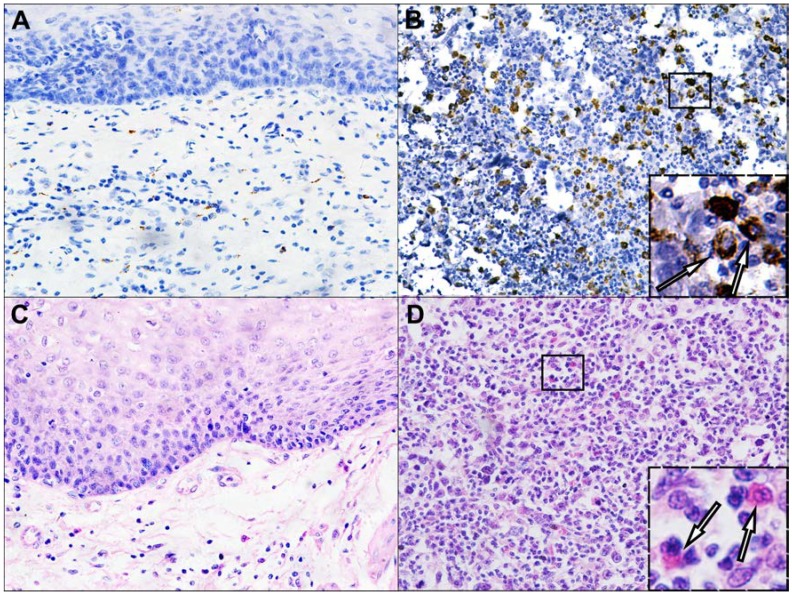
Infiltration of macrophages and eosinophils into the SmCEC tissues and tumor adjacent normal tissues. (**A**) Macrophages in tumor adjacent normal tissue; (**B**) Macrophages in SmCEC; (**C**) Eosinophils in tumor adjacent normal tissue; and (**D**) Eosinophils in SmCEC. (Original magnification 200×) Insets: higher magnifications of macrophages and eosinophils (Original magnification 800×).

Of the different tumor infiltrating inflammatory cells analyzed, macrophages were found to be significantly increased in tumor tissues (*p* < 0.001) compared to tumor adjacent normal tissues (Table 1, Figure 1A,B). Similarly, eosinophils were also significantly increased in SmCEC tumor tissues (*p* < 0.001) in comparison with tumor adjacent normal tissues (Table 1, Figure 1C,D). No significant differences were observed in the average number of neutrophils and lymphocytes between SmCEC tissues and tumor adjacent normal tissues.

**Table 1:** Inflammatory-cell infiltration in SmCEC (small cell esophageal carcinoma) and in tumor-adjacent normal tissues.

Inflammatory Cell Type	Cell Count (Mean ± SD)/HPF	*p*
*Eosinophils*		
SmCEC (*n* = 36)	7.57 ± 4.63/HPF	<0.001
Normal (*n* = 19)	2.44 ± 2.46/HPF	-
*Neutrophils*		
SmCEC (*n* = 36)	1.93 ± 1.14/HPF	0.095
Normal (*n* = 19)	1.39 ± 0.52/HPF	-
*Lymphocytes*		
SmCEC (*n* = 36)	89.64 ± 23.90/HPF	0.199
Normal (*n* = 19)	78.94 ± 17.23/HPF	-
*Macrophages*		
SmCEC (*n* = 36)	15.12 ± 8.53/HPF	<0.001
Normal (*n* = 19)	5.25 ± 4.23/HPF	-

Abbreviations: HPF, High-Power Field.

### 2.3. Eosinophils and Macrophages Are Increased in ESqCC (Esophageal Squamous Cell Carcinoma) Tissues in Comparison with SmCEC Tissues

Eosinophils were observed in a significantly higher number in ESqCC tissues in comparison with SmCEC tissues (*p* = 0.038, Table S2). Macrophages were significantly increased in ESqCC tissues in comparison with SmCEC tissues (*p* < 0.001, Table S2). The average number of neutrophils and lymphocytes between ESqCC tissues and SmCEC tissues were not significantly differed (*p* > 0.05, Table S2).

### 2.4. Correlation of Inflammatory Cells with Clinico-Pathological Characteristics of SmCEC Patients

The median value of each inflammatory cell count was used as a cut-off to divide patients into two groups namely, the high infiltration group (above the median value) and the low infiltration group (below or equal to the median value). Correlation of each inflammatory cell count with clinico-pathological characteristics of patients revealed that, only the macrophage cell count was significantly associated (*p* = 0.015, Table 2) with lymph node metastasis.

**Table 2:** Correlation of inflammatory-cell infiltration with clinicopathologic features of the 36 SmCEC patients.

Features	Macrophages				Eosinophils					Neutrophils						Lymphocytes			
	Low		High	*p*	Low	High		*p*		Low		High		*p*		Low	High		*p*
*Sex*																			
Male	12		13	1.000	12	13		1.000		12		13		1.000		13	12		1.000
Female	6		5	-	6	5		-		6		5		-		5	6		-
*T stage*																			
T1 + T2	9		13	0.305	12	10		0.733		11		11		1.000		12	10		0.733
T3 + T4	9		5	-	6	8		-		7		7		-		6	8		-
*pTNM stage*																			
I + II	9		15	0.075	13	11		-		13		11		0.725		13	11		0.725
III + IV	9		3	-	5	7		-		5		7		-		5	7		-
*Location of the primary tumor*																			
Lt	5		5	1.000	5	5		1.000		5		5		0.273		5	5		1.000
Mt	12		11	-	12	11		-		10		13		-		12	11		-
Ut	1		2	-	1	2		-		3		0		-		1	2		-
*Macroscopic tumor type*																			
Ulcerative	8		6	0.345	8	6		0.922		8		6		0.150		7	7		0.056
Medullary	8		6	-	7	7		-		4		10		-		4	10		-
Intraluminal	0		3	-	1	2		-		3		0		-		3	0		-
Mushroom	2		3	-	2	3		-		3		2		-		4	1		-
*N stage*																			
N0	3		11	0.015 *	5	9		0.305		7		7		1.000		7	7		1.000
N1+N2	15		7	-	13	9		-		11		11		-		11	11		-
*Age, years*																			
≤60	10		9	1.000	9	10		1.000		11		8		0.505		12	7		0.181
>60	8		9	-	9	8		-		7		10		-		6	11		-
*Tumor size, cm*																				
≤4	8		12	0.315	13	7		0.092		10		10		1.000		10	10		1.000
>4	10		6	-	5	11		-		8		8		-		8	8		-

Abbreviations: T stage, pathologic stage based on tumor size; N stage, pathologic stage based on lymph node involvement; Lt, lower thoracic esophagus; Mt, middle thoracic esophagus; Ut, upper thoracic esophagus; * *p* < 0.05. TNM staging: A cancer staging system in which T describes the size of the primary tumor and whether it has invaded nearby tissue, N describes regional lymph nodes involvement, and M describes distant metastasis.

### 2.5. Survival Analyses

As of 30 June 2013, survival of 36 patients after the initial treatment ranged from two to a maximum of 143 months. The median survival time was 22 months. The one year and five year survival rates were 66.67% and 5.56% respectively. Only two patients survived more than 5 years, both of whom underwent surgery followed by postoperative radiotherapy and/or chemotherapy.

### 2.6. Inflammatory Cell Counts Associated with Survival in SmCEC Patients

**Figure 2:**
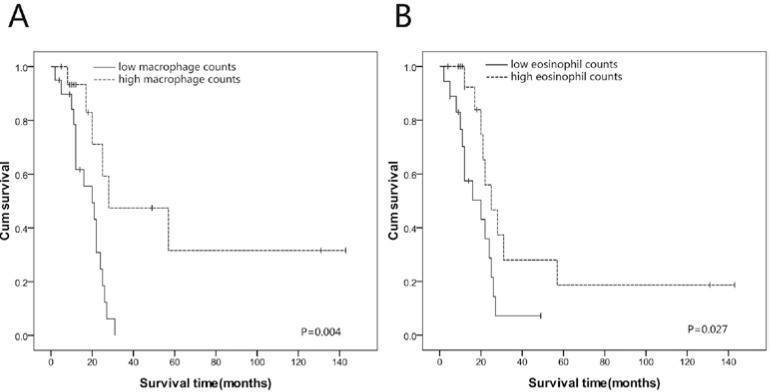
Kaplan-Meier survival curves of SmCEC patients (**A**) High macrophage counts in SmCEC correlated with better overall survival (*p* = 0.004); (**B**) High eosinophil counts in SmCEC correlated with better overall survival (*p* = 0.027).

Using the median value of each inflammatory cell, patients were divided into low infiltration group (below or equal to median) and high infiltration group (above the median value). Prognostic significance of low *vs.* high infiltration groups for each inflammatory cell was analyzed through Kaplan-Meier method. Patients in the high macrophage infiltration group had significantly prolonged overall survival in comparison with low macrophage infiltration group (*p* = 0.004, Figure 2). Similarly, patients of high eosinophil infiltration group experienced significantly better survival (*p* = 0.027, Figure 2) when compared with patients in the low eosinophil infiltration group. Nonetheless, other inflammatory cells such as neutrophils and lymphocytes did not show a significant association with overall survival (*p* > 0.05).

Univariate analysis was performed and on performing this analysis, age, sex, TNM stage, tumor size, lymph node metastasis, depth of invasion, tumor location and tumor type showed no correlation with prognosis. A high macrophage count (*p* = 0.008), high eosinophil count (*p* = 0.036) and chemotherapy (*p* = 0.003) were significantly associated with prolonged survival (Table 3). Multivariate analysis predicted that, high eosinophil count (*p* = 0.002) and chemotherapy (*p* = 0.001) were independent prognostic indicators (Table 3). Furthermore, patients who received postoperative chemotherapy had a relatively prolonged survival compared to patients who did not receive postoperative chemotherapy (data not shown).

**Table 3:** Univariate and multivariate Cox regression analysis for the prognosis of the 36 SmCEC patients.

Feature	Univariate Analysis		Multivariate Analysis	
	Hazard Ratio (95% CI)	*p*	Hazard Ratio (95% CI)	*p*
*Macrophage counts*				
Low *vs.* High	0.247 (0.088–0.693)	0.008	-	-
*Eosinophil counts*				
Low *vs.* High	0.386 (0.159–0.938)	0.036	0.209 (0.078–0.559)	0.002
*Chemotherapy*				
Yes *vs.* No	0.239 (0.092–0.623)	0.003	0.133 (0.045–0.395)	0.001
*pTNM stage*				
I + II *vs.* III + IV	1.139 (0.337–3.836)	0.834	-	-
*Tumor size*				
≤4 cm *vs.* >4 cm	1.096 (0.318–3.852)	0.884	-	-
*Lymph node metastasis*				
N0 *vs.* N1 + N2	0.847 (0.271–2.653)	0.776	-	-
*Depth of invasion*				
T1 + T2 *vs.* T3 + T4	1.526 (0.476–4.894)	0.477	-	-
*Tumor location*				
Mt *vs.* Ut + Lt	1.326 (0.618–2.844)	0.468	-	-
*Macroscopic tumor type*				
Ulcerative + Medullary *vs.* Intraluminal + Mushroom	1.082 (0.551–2.124)	0.820	-	-
*Age*				
≤60 years *vs.* >60 years	0.912 (0.283–2.938)	0.877	-	-
*Sex*				
Male *vs.* Female	0.470 (0.136–1.628)	0.234	-	-

Abbreviations: CI, confidence interval.

### 2.7. Discussion

In this study, we observed a significant infiltration of macrophages and eosinophils into the SmCEC tumors in comparison with tumor adjacent normal tissues. Likewise, infiltration of macrophages and eosinophils were significantly increased in ESqCC tumors than in SmCEC tumors. Of the different inflammatory cells, only macrophage infiltration was significantly associated with lymph node metastasis. Kaplan-Meier analysis unveiled that, patients with high macrophage infiltration and high eosinophil infiltration had prolonged overall survival than patients with low macrophage and low eosinophil infiltration. Multivariate Cox regression analysis showed that, number of eosinophils and chemotherapy were independent predictors for survival. 

We observed a significantly increased infiltration of macrophages and eosinophils into SmCEC tumors than in tumor adjacent normal tissues. The reason for this increase over the normal tissue is due to the fact that, tumor cells and stromal cells (fibroblasts, endothelial cells, macrophages) secrete a number of chemo-attractants which in turn recruit monocytes from the blood stream to the sites of inflammation where they differentiate into macrophages. For instance, high levels of CCL2 (a chemotactic protein) correlated with increased number of macrophages in many human tumors [32]. Other chemokines reported to recruit macrophages are CCL3, CCL4, CCL5, CCL7, CCL8, CXCL12, and IL-10 [33,34,35,36]. In addition, high mobility group box protein 1 released by dying tumor cells found in the necrotic areas of tumors have also been reported to recruit monocytes [37]. Concerning eosinophils, they are implicated in the pathogenesis of numerous inflammatory processes including cancer. Studies on several human tumors including oral squamous cell carcinoma [38], gastrointestinal tumors [39], Hodgkin lymphoma [40], and nasopharyngeal tumors [41], reported increased number of eosinophils. Mechanism of eosinophil recruitment into the tumor is well established. Eosinophils express CCR3 (CC Chemokines Receptor 3), a major eosinophil chemokine receptor and eotaxin (CCL11) is a chemokine that binds to the CCR3 and mediates the recruitment of eosinophils to the tumor microenvironment [42]. 

We also compared the mean number of inflammatory cells observed in SmCEC with ESqCC and found that macrophages and eosinophils were significantly increased in ESqCC. Previous studies have shown that, macrophages [12], and eosinophils [16], were infiltrated into ESqCC. Yet, no studies compared these cells between SmCEC and ESqCC and on comparison; macrophages and eosinophils were almost doubled in average number in ESqCC. This clearly suggested to us that the degree of infiltration of inflammatory cells differs between these two histological sub-types of esophageal carcinoma.

In this study, patients belonging to the low macrophage infiltration group often experienced more regional lymph node metastases than patients in the high macrophage infiltration group. Gulubova *et al.* [43] showed that, metastases to local lymph nodes significantly correlated with lower number of macrophage (CD68) cells in colorectal cancer. Macrophages based on their activation state divide into M1 and M2 cells. M1 cells are anti-tumorigenic whereas M2 cells are pro-tumorigenic. Several lines of evidence demonstrate that in non-progressing or regressing tumors, macrophages exhibit M1 phenotype, whereas, in malignant tumors, macrophages exhibit the M2 phenotype [44,45]. Kurahara *et al.* [46] showed that, a high number of M2 macrophages resulted in high incidence of lymph node metastases in pancreatic cancer. Algars *et al.* [47] reported that, the function of macrophages appears to vary during the progression of colorectal cancer. During the earlier stage, macrophages may mediate an anti-tumor response, though at a later point of this stage, a small sub-population of suppressive macrophages is not capable of shifting the immune balance to a suppressive trend. Contrastingly, in an advanced stage, an increased number of M2 macrophages promote tumor growth and metastasis. This mechanism might explain our observation in this study that, low macrophage infiltration leads to lymph node metastasis, in other words, as the tumor progress, macrophages are polarized to the M2 type which causes the tumor to metastasize to lymph nodes. However, to confirm this, we need to analyze specific macrophage markers in N0, N1, N2 and N3 SmCEC tumor tissues. 

In this study, patients belonging to the high macrophage infiltration group experienced significantly increased overall survival than patients in low macrophage infiltration group. Similar to our study, Forssell *et al.* [48] reported that, patients with high macrophage infiltration had significant survival advantage in colorectal cancer. Gulubova *et al.* [43] reported that, patients with low macrophage infiltration had significantly shorter survival compared with high macrophage infiltration. Oberg *et al.* [49] reported that, the 5-year survival rate for patients with high CD68 was 60%, whereas, the same was 38% for those with low CD68. It has been suggested that, tumors that grow in the gastrointestinal tract possess a large number of microflora, so there is high synthesis of pro-inflammatory cytokines directed against these microflora [50,51], and this antimicrobial immune response becomes simultaneously anti-tumorigenic [43]. Furthermore, in an *in vitro* study [48], phorbol 12-myristate 13-acetate (PMA) differentiated UM937 cells (a cell line of monocytic origin that can be induced to differentiate into macrophage-like cells upon treatment with PMA) inhibited HCT-116 colon cancer cell survival and this inhibition was dependent on the density of PMA-activated U937 cells to colon cancer cells in a 10:1 ratio. Thus, a high macrophage infiltration into the tumor is believed to result in high macrophage to cancer cell ratio, which might kill tumor cells and thereby improve the prognosis of patients in our study.

However, the role of macrophages in prognosis is still controversial. Several clinical studies reported that, macrophage infiltration was associated with both good prognosis [48,52,53], and poor prognosis [54,55]. This discrepancy is due to that, almost, if not all of these studies employed CD68, a pan-macrophage marker that is recognized by both M1 tumoricidal and M2 anti-inflammatory macrophages. Furthermore, the activity (anti-tumor/pro-tumor) of macrophages depends on various micro-environmental factors affecting the polarization and functionality of these macrophages. However, further investigation with an M1 specific marker is required to confirm whether increased macrophage infiltration would improve the survival of SmCEC patients in this study.

In the current study, eosinophils were also associated with survival; a high eosinophil infiltration into the tumor exerts a favorable effect on survival. Electron microscopic studies have provided evidence for the existence of interaction between eosinophils and tumor cells in gastric cancer [56]. Clinical studies have observed that, a high eosinophil infiltration into the tumor was significantly associated with improved prognosis in patients than that with low eosinophil infiltration in ESqCC [13], in advanced gastric cancer patients [57], and in colorectal cancer [58]. 

Eosinophils contain cytotoxic granular proteins and upon activation secrete many cytokines that kill tumor cells. Interleukin-5 secreted by stromal cells of the tumor activates eosinophils, which in turn liberate toxic granules to exert cytotoxic effects on tumor cells [59]. Interleukin-2 treatment in patients significantly elevated the levels of cytotoxic granular proteins [60]. An ultra-structural study has identified degranulated eosinophils and numerous extracellular granules in the tumor stroma of gastric carcinoma patients [57]. Furthermore, these extracellular granules are capable of acting as a functional “minefield” amplifying the differential secretory properties of eosinophils and thereby adding to the persistence and exacerbation of the inflammatory response in the tumor stroma [61]. Thus, the improved survival observed for patients in the high eosinophil infiltration group may be due to the above-mentioned anti-tumorigenic effects of eosinophils. 

In this study, macrophage count, eosinophil count and chemotherapy were found to be associated with survival in a univariate analysis. However, in the multivariate analysis only eosinophil count and chemotherapy turned out to be the independent prognostic indicators whereas, the macrophage count did not remain as a significant parameter in predicting patients’ survival. This may be as a result of CD68 pan-macrophage marker that we used in this study, which could stain both antitumor (M1) and pro-tumor (M2) macrophages. Eosinophils as an independent prognostic indicator of survival can be explained by the fact that, tumor stromal cells activate eosinophils which in turn secrete cytotoxic granular proteins such as eosinophil peroxidase, and eosinophil cationic protein *etc.*, which in turn act on tumor cells and directly kill them [62]. Lv *et al.* [1] reported that, SmCEC is primarily sensitive to chemotherapy or radiotherapy. Our result about chemotherapy as an independent prognostic indicator for improved survival was consistent with a previous study, in which median survival time for patients who received chemotherapy was longer than systemic therapy. In addition to this, the same study found that, chemotherapy was an independent prognostic factor in multivariate analysis in SmCEC. 

Infrequently, small cell carcinoma manifest outside the lungs and pleural space. Primary sites include esophagus, salivary gland, cervix, prostate, pancreas, gastrointestinal tract and skin [63]. Since, the clinical symptoms and tumor behaviour of SmCEC are similar to ESqCC and small cell cancer respectively; we compared the effects of tumor infiltrating inflammatory cells in SmCEC of the present study with the data in published studies in ESqCC and small cell lung cancer (Supplementary Information, Table S3). In these studies, a high macrophage count proved to be a prognostic marker in predicting favorable survival for small cell lung cancer patients after surgery [26], and increased macrophage infiltration seemed to be associated with poorer survival rates in ESqCC patients [12]. Our findings on macrophages in the present study are consistent with small cell lung cancer, but are in contrast to that in ESqCC. However, our findings on eosinophils in the present study are similar to those reported by Ishibashi *et al.* [64] in ESqCC where they found that the infiltration of a large number of eosinophils resulted in a better prognosis. 

Immune cells such as neutrophils and lymphocytes have been previously associated with prognosis in cancer patients. For instance, Klintrup *et al.* [65] found that lymphocyte count in the intra-tumoral region has significant prognostic value and also a high neutrophilic granulocyte number at the invasive margin indicated a good prognosis. In our study cohort, neutrophils and lymphocytes had no apparent effect on prognosis. Further studies with larger number of samples with more sensitive methods of detection are needed to answer this complex question. 

Our study has some strengths and limitations. Sample sizes of previous studies in SmCEC described in the literature have been usually less with many of those in the form of case reports. But, we investigated inflammatory cell infiltration in 36 SmCEC patients. We have included cancer patients with all four stages (Stage I, II, II and IV) in this study. More importantly, our study is the first report on the prognostic influence of inflammatory cells in SmCEC. A major limitation of our study though is the usage of CD68, a pan-macrophage marker that has been found to recognize both M1 and M2. 

## 3. Experimental Section

### 3.1. Patients and Tissue Collection

In this retrospective cohort study, a total of 36 SmCEC specimens along with 19 tumor adjacent normal tissue specimens were selected from patients who visited the cancer hospital of Shantou University Medical College and the central hospital of Kaifeng during the period from November 1997 through March 2013. A total of 16 ESqCC histologically confirmed tumor specimens were also collected for this study.

SmCEC was diagnosed in all 36 patients by Hematoxylin and Eosin staining of tumor tissue specimens and confirmed by detection of neuroendocrine markers such as neuron-specific enolase, synaptophysin and chromogranin-A. Two expert pathologists histologically confirmed that tissue specimens were SmCEC. Routine physical examinations included chest radiographs and CT scans and revealed no evidence of other tumors, including small cell lung cancer, and none of the patients underwent chemotherapy or radiotherapy before surgery or biopsy. Patients were staged according to the American Joint Committee on Cancer (7th edition) classification. The Institutional Review Board and the Ethics committee of the Cancer Hospital of Shantou University Medical College approved this study.

### 3.2. Morphological Identification and Enumeration of Eosinophils, Neutrophils and Lymphocytes

Immediately after surgical resection, tumor tissues were fixed in 10% formalin buffered with phosphate (pH 7.4) and subsequently embedded in paraffin wax. Paraffin blocks with tumor tissue mounted were cut into 4 µm thickness sections and subsequently stained using Hematoxylin and Eosin. Nucleated cells with intensely red cytoplasmic granules were accepted as eosinophils and care was taken to exclude red blood cells with superimposed mononuclear and polymorphonuclear inflammatory cells. Granular leukocytes having a nucleus with three to five lobes connected by threads of chromatin, and cytoplasm containing very fine granules were considered as neutrophils. Lymphocytes comprised foci with mononuclear leukocytes having deeply stained nucleus containing dense chromatin and pale blue-stained cytoplasm. Inflammatory cells infiltrated into the tumor stroma were taken for analysis and inflammatory cells confined to lymph vascular spaces or within the vicinity of tumor necrosis or secretions were excluded from analysis. Inflammatory cells were then scored in 10 non-overlapping high power fields (400×) in each specimen and the average number was taken for analysis. 

### 3.3. Immunohistochemistry and Evaluation of CD68 (A Pan-Macrophage Marker)

The formalin fixed, paraffin embedded tumor tissues were cut into serial sections of 4 µm thickness. These sections were deparaffinised and rehydrated by transfer through graded concentrations of ethanol to distilled water. Thereafter endogenous peroxidase activity was blocked by treating sections in 3% H_2_O_2_ for 30 min at room temperature. Then, antigen retrieval was performed in a microwave containing Tris-EDTA buffer (10mM Tris, 1 mM EDTA, pH 9.0) for 30 min, subsequently; sections were incubated in 10% normal serum for 30 min at room temperature to block non-specific binding of antibodies. Then, sections were incubated with CD68 antibody (1:300 dilution; Zhongshan Golden Bridge Biotechnology Company, Beijing, China) at 4 °C for 24 h. Then, the sections were incubated with horseradish peroxidase-conjugated mouse anti-rabbit secondary antibody (Dako Japan Ltd., Tokyo, Japan) at 37 °C for 30 min. Chromogenic detection of CD68 was performed with 0.02% of 3,3'-diaminobenzidine and 0.005% of H_2_O_2_ in 0.05 mmol/L Tris-HCl buffer and counter stained with hematoxylin. Sections immunostained with mouse IgG as the primary antibody were used as negative controls. To enumerate the number of CD68+ macrophage, “hot spots”, which are characterized by a high density of CD68+ macrophages, initially identifications by scanning sections at a lower magnification level (100×) were made. CD68+ macrophages were scored from 10 non-overlapping high power fields at 400× magnification and the average number was taken for analysis.

### 3.4. Enumeration of Inflammatory Cells in ESqCC

We also examined eosinophils, neutrophils, lymphocytes and macrophages in the same manner as described above, in 16 ESqCC tissue specimens. All the 16 ESqCC patients did not undergo chemotherapy or radiotherapy prior to surgery or biopsy. We included these samples merely to know whether any significant difference exists in inflammatory cell counts between SmCEC and ESqCC. 

### 3.5. Statistical Analysis

All statistical analyses were performed using SPSS software (IBM, Armonk, NY, USA). The Mann-Whitney U test was applied to find significant differences in the average number of each inflammatory cell between SmCEC and tumor adjacent normal tissues and between ESqCC and SmCEC. Fisher’s exact probability test was employed to find significant relationships between the average number of each inflammatory cell and clinico-pathological characteristics of patients. Clinico-pathological characteristics included are age (≤60 years *vs.* >60 years) sex, length of primary lesion (≤4 cm *vs.* >4 cm), location of primary lesion (upper, mid, or lower esophagus), macroscopic tumor type, pT, pN, and pTNM stages. Cumulative patient survival was calculated using the Kaplan–Meier method and log-rank test was used to compare survival. Survival time was calculated from the start of treatment to the point of death or to the last follow-up contact. The prognostic significance of the clinico-pathological characteristics was determined using univariate Cox regression analysis. A Cox regression model for multivariate analysis was employed for factors that achieved significance in the univariate analysis. A *p*-value of less than 0.05 was considered to be significant and all tests were two-sided.

## 4. Conclusions

An increased number of macrophages and eosinophils were strongly associated with longer survival time in SmCEC patients. A low macrophage count was associated with metastasis of the tumor to regional lymph nodes. Of all the clinicopathologic features studied, only eosinophil count and chemotherapy were independent prognostic indicators in SmCEC patients. Future studies on tumor-infiltrating inflammatory cells in SmCEC will benefit an increased understanding of the disease, and promote the discovery of biomarkers and new treatment strategies for these patients.

## Supplementary Files

Supplementary File 1 Supplementary Information (PDF, 114 KB)
