# Supplementary material for: Clinical Impact of Tumor-Infiltrating Inflammatory Cells in Primary Small Cell Esophageal Carcinoma
Source: Int J Mol Sci. 2014 May 30;15(6):9718–34. doi: 10.3390/ijms15069718 (PMC4100116; doi:10.3390/ijms15069718)
Supplement: Supplementary File 1 — Supplementary Information (PDF, 114 KB) [file ijms-15-09718-s001.pdf]

Supplementary Information

Table S1. Patients' demographics, clinico-pathological characteristics and treatment.

Characteristic		No. of Patients (n = 36)
Gender	Male	25
	Female	11
Age, years		59 (45–77)
Location of tumor	Ut	3
	Mt	23
	Lt	10
Treatment	S	24
	S + C	9
	S + C + R	3
Median survival time, months		22 (2–143)

Abbreviations: Lt, lower thoracic esophagus; Mt, middle thoracic esophagus; Ut, upper thoracic esophagus; S, surgery; C, chemotherapy; R, radiotherapy.

Table S2. Inflammatory-cell infiltration in SmCEC (small cell esophageal carcinoma) and ESqCC (esophageal squamous cell carcinoma).

Inflammatory Cell Type	Cell Count (Mean ± SD)/HPF	p
Eosinophils		
SmCEC (n = 36)	7.57 ± 4.63/HPF	0.038
ESqCC (n = 16)	13.15 ± 7.41/HPF	-
Neutrophils		
SmCEC (n = 36)	1.93 ± 1.14/HPF	0.071
ESqCC (n = 16)	2.31 ± 0.68/HPF	-
Lymphocytes		
SmCEC (n = 36)	89.64 ± 23.90/HPF	0.286
ESqCC (n = 16)	100.11 ± 39.85/HPF	-
Macrophages		
SmCEC (n = 36)	15.12 ± 8.53/HPF	<0.001
ESqCC (n = 16)	28.12 ± 7.91/HPF	-

Abbreviations: HPF, High-Power Field.

Table S3. Tumor-infiltrating inflammatory cells in small cell lung cancer, ESqCC and SmCEC.

Inflammatory Cell Type	Small Cell Lung Cancer	ESqCC	SmCEC in this Study
Macrophages	A high number of intratumoral macrophages was associated with a favorable survival [26]	The 5-year survival rate of patients with high MϕI total was significantly lower than that of patients with low MϕI total [12]	High macrophage counts were correlated with better overall survival
Eosinophils	No related information	Cases associated with a larger number of tumor-associated eosinophil infiltrations usually demonstrated better clinical outcomes [13]	High eosinophil counts were correlated with better overall survival
Lymphocytes	A high number of intratumoral T cells was associated with a significantly better survival time [26]	Dense tumor infiltration by UCHL-1-positive T-cells within the cancer cell nests was related to a better prognosis [65]	No statistical significance
Neutrophils	No related information	No related information	No statistical significance

© 2014 by the authors; licensee MDPI, Basel, Switzerland. This article is an open access article distributed under the terms and conditions of the Creative Commons Attribution license (http://creativecommons.org/licenses/by/3.0/).
